# Usefulness of the MAGGIC Score in Predicting the Competing Risk of Non-Sudden Death in Heart Failure Patients Receiving an Implantable Cardioverter-Defibrillator: A Sub-Analysis of the OBSERVO-ICD Registry

**DOI:** 10.3390/jcm11010121

**Published:** 2021-12-27

**Authors:** Marco Canepa, Pietro Palmisano, Gabriele Dell’Era, Matteo Ziacchi, Ernesto Ammendola, Michele Accogli, Eraldo Occhetta, Mauro Biffi, Gerardo Nigro, Pietro Ameri, Giulia Stronati, Italo Porto, Antonio Dello Russo, Federico Guerra

**Affiliations:** 1 Cardiovascular Disease Unit, IRCCS Ospedale Policlinico San Martino, IRCCS Italian Cardiovascular Network, 16100 Genova, Italy; marco.canepa@unige.it (M.C.); pietro.ameri@unige.it (P.A.); italo.porto@unige.it (I.P.); 2 Department of Internal Medicine, University of Genova, 16100 Genova, Italy; 3 Division of Cardiology, Cardinale G. Panico Hospital, 73039 Tricase, Italy; dr.palmisano@libero.it (P.P.); accogli.michele@libero.it (M.A.); 4 Division of Cardiology, Hospital Maggiore Della Carità, 28100 Novara, Italy; gdellera@gmail.com (G.D.); eraldo864@gmail.com (E.O.); 5 Institute of Cardiology, University Hospital Policlinic S. Orsola-Malpighi, 40121 Bologna, Italy; matteo.ziacchi@gmail.com (M.Z.); mauro.biffi@aosp.bo.it (M.B.); 6 Division of Cardiology, Vincenzo Monaldi Hospital, 80100 Naples, Italy; ammendolaernesto@libero.it (E.A.); gerardo.nigro@unicampania.it (G.N.); 7 Cardiology and Arrhythmology Clinic, Marche Polytechnic University, 60121 Ancona, Italy; g.stronati@staff.univpm.it (G.S.); a.delllorusso@staff.univpm.it (A.D.R.)

**Keywords:** competing risk, ICD therapies, implantable cardioverter-defibrillator, sudden cardiac death

## Abstract

The role of prognostic risk scores in predicting the competing risk of non-sudden death in heart failure patients with reduced ejection fraction (HFrEF) receiving an implantable cardioverter-defibrillator (ICD) is unclear. To this goal, we evaluated the accuracy and usefulness of the Meta-Analysis Global Group in Chronic Heart Failure (MAGGIC) score. The present analysis included 1089 HFrEF ICD recipients enrolled in the OBSERVO-ICD registry (NCT02735811). During a median follow-up of 36 months (1st–3rd IQR 25–48 months), 193 patients (17.7%) experienced at least one appropriate ICD therapy, and 133 patients died (12.2%) without experiencing any ICD therapy. The frequency of patients receiving ICD therapies was stable around 17–19% across increasing tertiles of 3-year MAGGIC probability of death, whereas non-sudden mortality increased (6.4% to 9.8% to 20.8%, *p* < 0.0001). Accuracy of MAGGIC score was 0.60 (95% CI, 0.56–0.64) for the overall outcome, 0.53 (95% CI, 0.49–0.57) for ICD therapies and 0.65 (95% CI, 0.60–0.70) for non-sudden death. In patients with higher 3-year MAGGIC probability of death, the increase in the competing risk of non-sudden death during follow-up was greater than that of receiving an appropriate ICD therapy. Results were unaffected when analysis was limited to ICD shocks only. The MAGGIC risk score proved accurate and useful in predicting the competing risk of non-sudden death in HFrEF ICD recipients. Estimation of mortality risk should be taken into greater consideration at the time of ICD implantation.

## 1. Introduction

Several prognostic risk scores have been developed to predict death in heart failure with reduced left ventricular ejection fraction (HFrEF) patients, with the Seattle Heart Failure Model (SHFM) being the most popular and validated [1]. More recently, the Meta-Analysis Global Group in Chronic Heart Failure (MAGGIC) risk score has been generated from a large European consortium including more than 39,000 heart failure (HF) patients [2] and proved more accurate than the SHFM [3]. Both scores comprise variables related to demographics, cardiac and non-cardiac comorbidities, and have been shown to possess a greater accuracy in predicting HF-related deaths (i.e., pump failure) and non-cardiovascular deaths rather than sudden cardiac death (SCD) [4,5].

Modes of death in HFrEF patients have been changing with time, with a significant reduction of SCD in the last decades largely explained by the widespread implementation of HF guideline-recommended therapies [6]. However, most implantable cardioverter-defibrillators (ICD) are today implanted for primary prevention of SCD in these patients [7], with or without an associated biventricular pacemaker according to guidelines. A growing number of reports emphasize the clinical relevance of the competing risk of non-sudden death in this population, particularly in the presence of comorbidities [8]. Overall, only about a quarter of these ICD recipients experience the ideal clinical course of receiving a protection from life-limiting ventricular arrhythmias and surviving for a reasonable period of time [9,10,11].

The overall scope of this analysis was to evaluate the accuracy and usefulness of the MAGGIC risk score in predicting the competing risk of non-sudden death in HFrEF patients receiving an ICD. We explored whether the probability of receiving appropriate potentially life saving ICD therapies or dying of causes other than SCD differed according to total mortality risk estimated using the MAGGIC risk score.

## 2. Material and Methods

The OBSERVational registry On long-term outcome of ICD patients (OBSERVO-ICD) was a multicenter, retrospective registry enrolling all consecutive patients from one of the five high-volume arrhythmia centers (Ancona, Tricase, Novara, Bologna, and Naples). In order to gather a “real-world” population, the only inclusion criteria were age ≥18 years and an ICD implant performed between 1 January 2010 and 31 December 2012. Patients had to be followed-up for at least three years to qualify for enrolment. The registry was endorsed by the Italian Association of Arrhythmology and Cardiac Pacing (AIAC) and registered into www.clinicaltrials.gov, accessed on 11 January 2021, (NCT02735811). All patients gave their written informed consent at the time of enrolment and the study was approved by the Ethic Committee of the proposing center. Detailed methods have already been published [12,13]. 

The present analysis focused on HFrEF, thus 192 patients with left ventricular ejection fraction (LVEF) > 40%, 14 patients with hypertrophic cardiomyopathy, 5 patients with arrhythmogenic cardiomyopathy, 2 patients with channelopathies were excluded from the original cohort of 1319 patients. Eighteen additional patients were lost at follow-up, leaving a final sample of 1089 individuals, of whom 96 (8.8%) were implanted for secondary prevention. 

### 2.1. Data Collection

At the time of ICD implantation complete medical history, laboratory markers, and device-related data were collected for all patients. A transthoracic echocardiogram was performed in all patients prior to device implant.

The MAGGIC score (www.heartfailurerisk.org, accessed on 11 January 2021) was calculated according to the final model by Pocock et al. [2] including the following 13 independent predictors of mortality: age, gender, EF, NYHA class, body mass index, serum creatinine, systolic blood pressure, time since HF diagnosis, diabetes, current smoking, chronic obstructive pulmonary disease, and current therapy with beta-blocker, ACE-inhibitor and angiotensin-receptor blockers. The score predicts the 1- and 3-year probability of all-cause death; this latter estimate was considered in the present analysis, considering a median length of follow-up of about 3 years (see below).

Data regarding ventricular arrhythmias and ICD therapies were collected through remote monitoring and in-office visit, as previously reported [12,13]. ICD therapies included all ventricular events requiring appropriate ICD intervention for ventricular tachycardia or ventricular fibrillation (including anti-tachycardia pacing, ATP). A parallel sensitivity analysis accounting only for ICD shocks but excluding ATP was conducted.

Survival status was documented through internal database network, ICD remote control, and direct phone interview. If a patient could not be contacted, the relatives or referring physicians were contacted instead in order to determine if the patient was deceased or readmitted to another hospital. Deaths were categorized into three predefined groups: non-sudden cardiac death (further categorized into HF death, coronary death, and other cardiac death), non-cardiac death, and sudden death. Cause of death was adjudicated by two authors by consensus, after careful examination of death certificates, clinical data and ICD interrogations, which were gathered post-mortem or through remote monitoring. No patients had their anti-tachycardia therapies deactivated or their device removed during follow-up.

The primary aim of this analysis was to evaluate the performance of the MAGGIC risk score in predicting different modes of death in a population of ICD recipients. To this goal, patients were censored at the time of death or first appropriate ICD therapy (or shock, in the sensitivity analysis), which was considered as proxy of SCD. Thus, the following events were included in the outcome variable: (1) non-sudden cardiac deaths and non-cardiac deaths composed the non-sudden death outcome; (2) ICD therapies (or shocks, in the sensitivity analysis) represented a proxy of SCD outcome. Secondary aim was to evaluate the potential role of the MAGGIC risk score in evaluating the competing risk of non-sudden death occurring prior of ICD therapy (or shock).

### 2.2. Statistical Analysis

Characteristics of patients at time of ICD implantation are presented as means and standard deviations, medians and interquartile ranges, or frequencies and percentages, as appropriate. ANOVA and Kruskal–Wallis were used to compare normally and non-normally distributed quantitative variables, respectively. A chi-squared test was used to compare categorical variables. The MAGGIC risk score was calculated according to the original model [2], and the frequency of different outcomes of interest was evaluated by tertiles of 3-year probability of death predicted by the MAGGIC score and compared using chi-squared test. ROC curves were obtained using logistic regression analysis, with the estimated MAGGIC probability of death tested separately against different outcomes (i.e., overall outcome, ICD therapy or shock, and non-sudden death). Area under the curves (AUC) and 95% confidence intervals were calculated and compared using independent ROC curves analysis. A competing risk analysis was also performed using cumulative incidence function (CIF) to estimate outcomes caused by ICD therapy (or shock) and non-sudden death in the presence of competing risks. Estimates for the cumulative incidence of these events were presented according to MAGGIC tertiles of 3-year probability of death. Gray’s test for equality of CIF was used to compare incidence between groups. Regressions on the CIF were calculated using the Fine–Gray model, obtaining sub-distribution hazard ratios (sHR) for each group and outcome of interest. All statistical analysis was performed using SAS 9.4 (SAS Institute, Cary, NC, USA). Values of *p* < 0.05 (two-tailed) were taken as statistically significant.

## 3. Results

One thousand and eighty-nine patients with HFrEF were included in the present analysis. Clinical characteristics of the population are shown in Table 1, overall and divided by MAGGIC tertiles of 3-year probability of death (low-risk < 23%, *n* = 345; mid-risk between 23% and 39%, *n* = 398; high-risk > 39%, *n* = 346). As expected according to the variables included in the score calculation, patients in the highest MAGGIC risk tertile were older, with lower systolic blood pressure, higher creatinine, and a greater burden of cardiac and non-cardiac comorbidities, including atrial fibrillation, diabetes and chronic obstructive pulmonary disease. In addition, their functional status was worse at the time of ICD implantation, they had lower left ventricular ejection fraction, a higher prevalence of ischemic etiology and more frequently received a biventricular ICD (Table 1).

During a median follow-up of 36 months (1st–3rd IQR 25–48 months), 193 patients (17.7%) experienced at least one appropriate potentially life-saving ICD intervention, of which 122 (63.2%) were ATP and 71 (36.8%) were shocks (14 preceded by ATP). Among these 193 patients, three experienced at least one appropriate ICD intervention before subsequently receiving a heart transplantation, which was performed in 25 patients overall during follow-up. One hundred and seventy-three patients died (15.9%), including two among those who received a heart transplantation during follow-up. Of all deaths, 72 (41.6%) were adjudicated as HF-related, five (2.9%) as due to acute coronary syndrome, 14 (8.1%) as related to cerebrovascular accidents, 35 (20.2%) as due to cancer, five (2.9%) as due to pulmonary disease, and 42 (24.3%) related to other non-cardiac causes. There were no sudden deaths in our population during follow-up. One hundred and thirty-three (77%) vs. forty deaths (23%) occurred respectively without and with receiving at least one appropriate ICD therapy (including 13 who received a shock, of whom two were preceded by ATP). Cause of death was HF-related in 60% of these patients and related to other non-cardiac causes in the remaining 40%. According to study protocol, these patients were censored at the time of first ICD therapy. Thus, in the final analysis there were 193 ICD therapies (14.0%) and 133 non-sudden deaths (12.2%, including 48 HF-related deaths and 85 other deaths), for a total of 326 (29.9%) overall outcomes of interest. This number decrease to 231 (21.2%) overall outcomes of interest when a less sensitive approach excluding ATP was used, leaving 71 ICD shocks (6.5%) and 160 non-sudden deaths (14.7%, including 64 HF-related deaths and 96 other deaths). Number of ICD interventions did not significantly differ between patients receiving an ICD in primary vs. secondary prevention (ICD therapies: primary 17.2% vs. secondary 22.9%, *p* = 0.16; ICD shocks: primary 6.7% vs. secondary 5.2%, *p* = 0.59).

The MAGGIC score predicted a median 3-year mortality of 31.6% (1st–3rd IQR 20.9–39.7), giving an observed to estimated mortality ratio of 0.95 (outcome with ICD therapy) and 0.67 (outcome with ICD shock only), suggestive of over-prediction in this population of HFrEF ICD recipients. 

**Figure 1:**
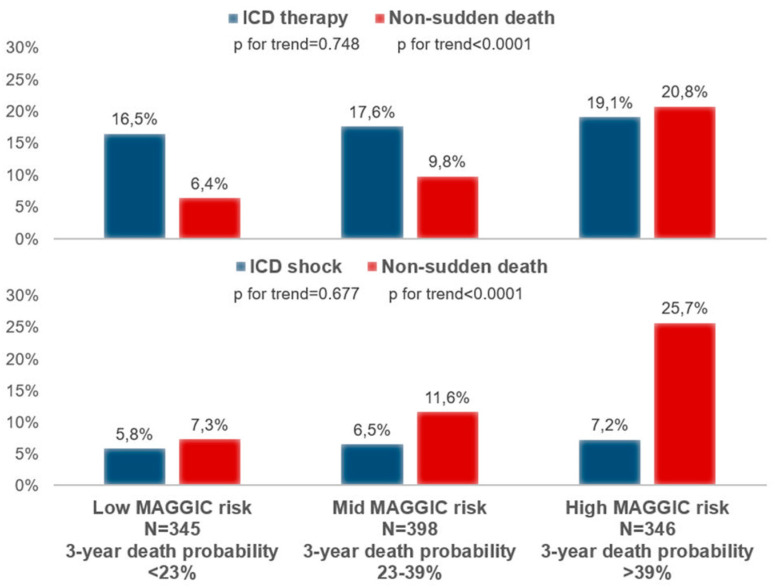
Frequency of ICD therapy or shock and non-sudden death by tertiles of 3-year death probability estimated using the MAGGIC score.

The frequency of patients receiving at least one ICD therapy during follow-up was substantially stable across increasing tertiles of 3-year death probability estimated using the MAGGIC score (from 16.5% to 17.6% to 19.1%, *p* = 0.748), whereas mortality increased with increasing tertiles of MAGGIC probability of death (from 6.4% to 9.8% to 20.8%, *p* < 0.0001, Figure 1). When ATPs were excluded from ICD therapies, results remained substantially unchanged, with ICD shocks across MAGGIC probability of death tertiles remaining substantially stable between 5.8% and 7.2% (*p* = 0.677) and non-sudden mortality steadily increasing (from 7.3% to 11.6% to 25.7%, *p* < 0.0001, Figure 1).

**Figure 2:**
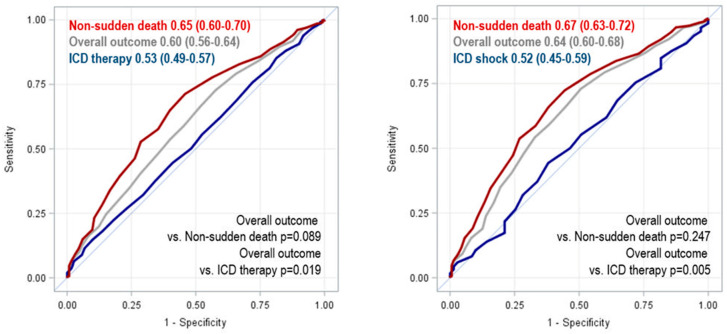
Accuracy of MAGGIC risk score in predicting ICD therapy or shock, non-sudden death, and the overall outcome.

Accuracy of MAGGIC score, expressed as ROC AUC, was 0.60 (95% CI, 0.56–0.64) for the overall outcome of ICD therapies and non-sudden deaths, 0.53 (95% CI, 0.49–0.57) for ICD therapies only and 0.65 (95% CI, 0.60–0.70) for non-sudden death only (Figure 2). When the same analysis was restricted to ICD shocks as the only proxy of SCD, results remained substantially unchanged; ROC AUC was 0.64 (95% CI, 0.60–0.68) for the overall outcome, 0.52 (95% CI, 0.45–0.59) for ICD shocks, 0.67 (95% CI, 0.63–0.72) for non-sudden death (Figure 2). 

**Figure 3:**
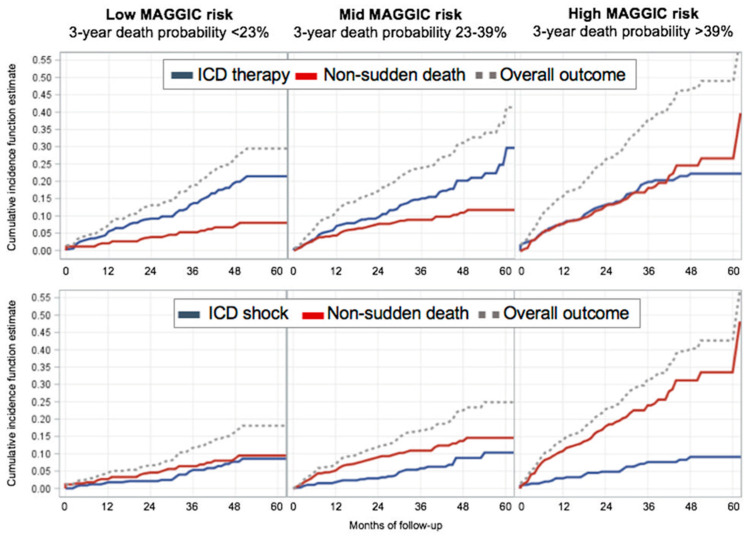
Cumulative incidence function estimate curves of competing causes of ICD therapy or shock and non-sudden death by tertiles of 3-year death probability estimated using the MAGGIC score.

In competing risk analysis (Figure 3), a significant effect on non-sudden death (*p* < 0.0001) but not on ICD therapy (*p* = 0.466) between the MAGGIC risk groups was found. Indeed, the hazard of non-sudden death for the high-risk MAGGIC group was 2.27-times higher than the one of the mid-risk group (95% CI 1.54–3.36) and 3.67-times higher than the one of the low-risk group (95% CI, 2.28–5.90). On the contrary, the hazard of ICD therapy for the high-risk MAGGIC group was statistically not greater than that of other groups (vs. mid risk sHR 1.25, 95% CI, 0.88–177; vs. low risk sHR 1.14, 95% CI, 0.82–1.60). Results were substantially unchanged when ICD shocks were considered in place of ICD therapies as proxy of SCD (non-sudden death: high vs. mid-risk sHR 4.08, 95% CI, 2.62–6.34; high vs. low-risk sHR 2.42, 95% CI, 1.70–3.46; ICD shock: high vs. mid-risk sHR 1.32, 95% CI, 0.73–2.36; high vs. low-risk sHR 1.13, 95% CI, 0.65–1.96). Whereas in the low-risk and mid-risk groups, cumulative incidence estimates of ICD therapies over the course of follow-up were two- to three-times higher than those of non-sudden death, in the high-risk group these estimates substantially matched (Figure 3 and Table 2). Notably, when accounting for ICD shocks only, cumulative incidence estimates of non-sudden death over the course of follow-up were two- to three-times higher than those of ICD shocks in both the mid-risk and high-risk groups starting from 12 months of follow-up (Figure 3 and Table 2).

## 4. Discussion

Our analysis confirmed a positive relationship between increased MAGGIC score and increased risk of non-sudden death in HFrEF patients implanted with an ICD, and the inaccuracy of this prognostic score in predicting ICD therapies. On the contrary, in competing risk analysis the MAGGIC score proved effective in identifying ICD recipients that were less likely to benefit of ICD therapies because of competing of non-sudden death.

This study reinforces previous evidence showing the scarce predictive value of available prognostic risk scores [4], and particularly the MAGGIC risk score [5], regarding SCD in HFrEF patients. We herein investigated a population of HFrEF ICD recipients and considered ICD therapies (or shocks) as proxies of SCD. The accuracy of the MAGGIC score in predicting these events was null, with a ROC AUC of 0.53 (95% CI, 0.49–0.57) for ICD therapies and of 0.52 (95% CI, 0.45–0.59) for ICD shocks. On the other hand, our findings confirmed the accuracy of this score in predicting the risk of non-sudden death in this population, and suggested its potential applicability in the definition of the competing risk of this outcome at the time of ICD implantation.

There has been considerable debate on this issue, particularly in the primary prevention setting [9], but few works to date have addressed it convincingly with appropriate competing risk analysis, which follows separate survival models estimating cause-specific hazard ratios for non-sudden death vs. ICD therapy [10,11,14,15]. In 2008, Koller and colleagues published a first landmark analysis of 442 ICD recipients who nonetheless were mostly implanted for secondary prevention (59% vs. 9% in our sample) and seldom received a biventricular pacemaker (16% vs. 44% in our sample) [10]. More recently, the same Authors reported on 720 HFrEF patients receiving a primary prevention ICD with a biventricular pacemaker [11]. Over a median follow-up of 7 years, 155 patients (22%) died prior to any ICD therapy, whereas 177 patients (24%) received at least one appropriate ICD therapy, which is comparable to our present analysis (16% and 18%, respectively, over a median follow-up of 3 years). Predictors of prior death as opposed to ICD therapy were older age, male gender, impaired renal function, low systolic blood pressure, obesity, peripheral arterial disease, and a history of cancer [11]. The role of these and other comorbidities in this setting has been increasingly acknowledged [8,16], and recent data suggest that up to 72% of primary prevention ICD recipients with three of more comorbidities die without prior ICD therapy [8]. To this regard, in a recent competing risk analysis, Poupin and colleagues evaluated mortality, appropriate ICD therapy rates and survival gain in 121 elderly patients (mean age 78 years, 83% male, 74% primary prevention, 51% with biventricular pacemaker) after risk stratification according to the Charlson Comorbidity Index (CCI). A CCI ≥ 4 was associated with a three-fold increased risk of 5-year all-cause mortality compared to a CCI ≤ 1, with the lowest cumulative incidence of first appropriate ICD therapy (7.3% at one year and 22.8% at five years, as compared to 11.6% and 34.2% in CCI ≤ 1) and potential gain of survival after a successful appropriate ICD intervention (1.4 years, as compared to >5 years in CCI ≤ 1) [14]. One- and five-year cumulative incidence estimates of ICD therapies were similar in our study (see Table 2), and not significantly modified after risk stratification according to MAGGIC tertiles of 3-year probability of death, ranging between 5.6% and 8.0% and between 21.5% and 29.7%, respectively. Of greater interest, however, was comparing these estimates with those of non-sudden death within each MAGGIC risk group. Whereas in the low-risk (3-year estimated mortality < 23%) and mid-risk (3-year estimated mortality 23–39%) groups, cumulative incidence estimates of ICD therapies over the course of follow-up were two- to three-times higher than those of non-sudden death, in the high-risk (3-year estimated mortality > 39%) group they substantially matched, supporting the increasing competing risk of non-sudden death evidenced by increasing MAGGIC risk score. More importantly, when ICD shocks only were considered, cumulative incidence estimates of non-sudden death over the course of follow-up were two- to three-times higher than those of ICD shocks in both the mid-risk and high-risk MAGGIC groups. To put this finding in context, a 75-year-old NYHA II male patient with 30% LVEF, 110 mmHg systolic blood pressure, 24 kg/m^2^ body mass index, 1.4 mg/dL creatinine, smoker with chronic obstructive pulmonary disease, without diabetes, and receiving treatment with both beta-blocker and renin-angiotensin system inhibitors would have a 3-year estimated mortality risk by MAGGIC score of 39.7%. Should this patient receive a primary prevention ICD? Our results suggest that by one year of follow-up after ICD implantation, this patient will have a 3% probability of having received a life-saving ICD shock, but an 11% probability of dying of non-sudden causes. These estimates will further diverge over time, and become 9% vs. 34% at five years of follow-up. We believe our work does not provide a definite answer to the above question, but offers a first ever evidence of the usefulness of the MAGGIC score in this context, and might further inform the dialogue between the clinician and the patient at the time of ICD implantation decision.

A similar approach implementing the use of prognostic models to predict the risk of non-sudden death without prior appropriate ICD intervention in patients with reduced LVEF was previously proposed by van Rees and colleagues [17], and subsequently by investigators of the Multicenter Automatic Defibrillator Implantation Trial (MADIT)-II [18] and of the Sudden Cardiac Death in Heart Failure Trial (SCD-HeFT), which developed a modified version of the SHFM to accommodate for variables available in the SCD-HeFT database [19]. In all these analyses, patients ranked in the highest-risk group displayed a >50% cumulative incidence of death without ICD interventions at long-term follow-up and no overall ICD survival benefit. Variables defining the highest mortality risk group in these three prognostic models included older age, male gender, lower systolic blood pressure, lower LVEF, and, higher NYHA functional class, history of diabetes mellitus and/or smoking, presence and severity of kidney disease, absence of HFrEF guideline-recommended therapy, which are all encompassed in the MAGGIC. A comparison among these models was recently performed using a single-center sample of 1969 primary prevention ICD recipients (average LVEF 29%, 58% with a biventricular pacemaker) followed-up for a median of 4.5 years (24% mortality, 68% of whom did not require any ICD intervention prior to death) [15]. Similarly to our results, accuracy of the three models in predicting non-sudden death ranged between 0.66 and 0.75, and the incidence of appropriate ICD interventions did not differ across the spectrum of risks, regardless of the model being used. Patients in the highest-risk group were more likely to experience ICD non-benefit than appropriate ICD intervention, and the best performance was obtained using the modified SHFM [15]. Unfortunately, the algorithm to calculate this latter score was not made public or translated into a web application like the MAGGIC.

Some previous works investigated ICD benefit in terms of the impact of ICD implantation on overall survival [20,21] rather than in terms of ICD therapy occurring prior of the competing event of non-sudden death, as we and others [10,11,14,15] did. Whereas in the latter case, the gain in survival can be attributed with great certainty to ICD intervention, in the first case, this is only presumed. Indeed, despite sophisticated statistical adjustments, unexplained confounding factors and unseen biases differentiating patients receiving or not an ICD cannot be completely ruled out. On the other hand, including ATP among life-saving ICD therapies, as done here and in previous works [14], may determine an overestimation of ICD benefit, because ventricular arrhythmias treated with ATP are typically different from those treated with shocks (i.e., lower ventricular rates, shorter arrhythmia onset to therapy) and may not be directly associated with fatal arrhythmias and SCD. This is why our analysis simultaneously reported and differentiated findings for ICD therapies and shocks, acknowledging the value of holding in higher consideration shocks than ATP, especially when performing competing risk analysis and using ICD interventions as proxies of SCD. Notably, the distribution of ATP vs. shocks at follow-up in our study (63% and 37%, respectively) was similar to the one of ICD interventions in the ventricular tachycardia (64%) vs. ventricular fibrillation zone (36%) in the study by Weber and colleagues [11].

A final comment regards key differences between prognostic scores discussed in our work. In particular, the accuracy of the SHFM was recently demonstrated inferior to that of other more recent prognostic scores, including the MAGGIC, due to an exaggerate reduction in the estimation of all-cause mortality risk by amplification of the SCD risk reduction obtained through ICD implantation [3]. Moreover, the MAGGIC score incorporates non-cardiac comorbidities such as chronic kidney disease and chronic obstructive pulmonary disease, which are not comprised in the SHFM, but are very pertinent to the purpose of the present analysis. Because of their major contribution to non-cardiovascular mortality, these two non-cardiac conditions, together with a history of cancer, are gaining larger attention in the HF cardiology community [11,22,23,24], and should be taken into greater account when estimating the competing risk of non-sudden death at the time of ICD implantation.

Some limitations of the present analysis should be acknowledged. This is a retrospective analysis performed on a multicentric database. Variables for the calculation of other prognostic scores, such as the SHFM, were missing in the database, thus comparisons between different scores could not be performed. The study sample size was limited and further reduced after stratification by MAGGIC risk, thus not allowing for the investigation of sub-groups of particular interest, such as patients without a biventricular pacemaker or receiving an ICD for secondary prevention. Because of the applicability of the MAGGIC score to the whole HFrEF spectrum, patients who received a secondary prevention ICD and/or with a LVEF between 36% and 40% were not excluded from the analysis. The addition of heart transplantation to non-sudden death deaths only determined an increase in the number of non-sudden competing event, without changing the overall significance of our findings. The ongoing collaboration between the five centers participating in the registry allowed for a homogeneous follow-up of the population, particularly regarding ICD programming and HF therapy [12,13]. Repeated or inappropriate ICD therapies were not considered in the present analysis and will be object of future specific investigations.

## 5. Conclusions

Prognostic risk scores conveniently combine demographics, cardiac and non-cardiac comorbidities in a unique number, and thus offer an integrated overall survival estimation that might be useful to capture the risk of non-sudden death in HFrEF ICD recipients. We herein demonstrated the accuracy and usefulness of the recently developed MAGGIC risk score to this goal. In patients estimated at higher 3–year mortality risk by using the MAGGIC score, the increase in the competing risk of non-sudden death over the entire course of follow-up was greater than that of receiving a first appropriate ICD therapy. These findings reinforce the importance and usefulness of performing survival risk estimation at the time of ICD implantation, with the ultimate goal of building a more informative dialogue between the clinician and the patient over expected ICD benefit.

## Figures and Tables

**Table 1:** General characteristics of the population at enrollment by categories of predicted 3-year mortality.

	Overall Sample *n* = 1089	Low MAGGIC Risk *n* = 345 3-Year Death Probability < 23%	Mid MAGGIC Risk *n* = 398 3-Year Death Probability 23–39%	High MAGGIC Risk *n* = 346 3-Year Death Probability > 39%	*p* Value
Age, years	66.1 ± 11.8	56.9 ± 11.0	66.7 ± 9.6	74.4 ± 7.9	<0.0001
Male sex, %	77.8	79.7	74.1	79.1	0.124
Systolic blood pressure (mmHg)	121.9 ± 16.9	124.5 ± 16.8	122.8 ± 17.0	118.3 ± 16.3	<0.0001
BMI (kg/m^2^)	26.3 ± 4.6	27.7 ± 4.9	26.1 ± 4.6	25.2 ± 4.0	<0.0001
Obesity	25.9	30.9	26.9	18.9	0.001
Dyslipidemia, %	64.7	61.0	63.6	67.9	0.154
COPD, %	26.8	11.4	27.9	40.4	<0.0001
NYHA					<0.0001
1	6.8	15.0	4.2	1.7	
2	51.9	73.0	53.4	28.9	
3	39.7	12.0	41.9	65.0	
4	1.6	0.0	0.5	4.3	
Current smoking	9.5	11.98	8.98	7.45	0.110
Hypertension	77.0	70.5	77.6	82.5	0.0007
Diabetes, %	34.4	17.8	34.2	50.1	<0.0001
Chronic kidney disease, %	24.8	9.8	23.4	41.3	<0.0001
Atrial fibrillation (paroxysmal or persistent), %	14.9	11.2	14.7	19.5	0.008
Atrial fibrillation (permanent), %	13.6	9.2	14.5	17.2	0.007
Left ventricular ejection fraction, %	28.4 ± 5.6	30.1 ± 5.5	28.1 ± 5.4	27.2 ± 5.5	<0.0001
HF diagnosis within 18 months, %	45.6	41.5	46.1	47.9	0.211
Creatinine (mg/dL)	1.2 ± 0.6	1.0 ± 0.4	1.2 ± 0.6	1.5 ± 0.8	<0.0001
Hemoglobin (g/dL)	12.9 ± 1.8	13.5 ± 1.7	12.9 ± 1.7	12.6 ± 2.0	<0.0001
Uric Acid (mg/dL)	6.4 ± 1.9	6.2 ± 1.8	6.4 ± 1.9	6.6 ± 2.2	0.004
Ischemic etiology, %	52.5	44.9	51.5	61.3	<0.0001
Previous CABG, %	23.3	17.1	21.1	32.1	<0.0001
Previous PCI, %	31.1	26.4	32.4	34.4	0.059
ICD type, %					0.001
Single chamber	24.2	29.6	19.6	24.3	
Dual chamber	31.8	34.8	32.7	27.8	
Biventricular	44.0	35.7	47.7	48.0	
Beta blockers, %	91.1	96.5	90.5	86.4	<0.0001
ACEi/ARB, %	88.7	91.9	88.7	85.6	0.031
Aspirin, %	59.6	58.8	59.1	60.7	0.86
Thienopyridines, %	18.7	13.7	21.1	21.0	0.022
Anticoagulant, %	30.1	22.6	30.7	36.8	0.0002
Diuretics, %	83.2	78.0	83.9	87.6	0.003
Antialdosterone drugs, %	57.6	53.9	56.8	62.1	0.084
Statins, %	64.5	61.2	66.6	65.5	0.274
Nitrates, %	17.6	12.2	17.1	23.7	0.0003
Amiodarone, %	21.6	17.4	21.9	25.4	0.036
Other antiarrhythmic drugs, %	1.7	0.6	1.3	3.5	0.01
Digitalis, %	11.2	8.4	13.6	11.3	0.084

BMI = body mass index; COPD = chronic obstructive pulmonary disease; NYHA = New York Heart Association; HF = heart failure; CABG = coronary artery bypass grafting; PCI = percutaneous coronary intervention; ICD = implantable cardioverter defibrillator; ACEi/ARB= angiotensin-converting enzyme inhibitor/angiotensin receptor blocker.

**Table 2:** Cumulative incidence estimates of outcomes of interest from competing causes according to tertiles of 3-year probability of death predicted by the MAGGIC score.

Follow-Up Time, Months	12	24	36	48	60
ICD therapy vs. non-sudden death					
	Low MAGGIC risk, *n* = 345				
Patients at risk	315	285	196	120	10
ICD therapy	5.6%	9.2%	13.7%	19.9%	21.5%
Non-sudden death	2.0%	3.9%	5.3%	7.3%	8.0%
	Mid MAGGIC risk, *n* = 398				
Patients at risk	352	310	207	101	13
ICD therapy	7.1%	9.7%	15.0%	20.2%	29.7%
Non-sudden death	4.3%	7.7%	8.9%	10.9%	11.7%
	High MAGGIC risk, *n* = 346				
Patients at risk	279	235	145	75	5
ICD therapy	8.0%	13.3%	19.9%	22.3%	22.3%
Non-sudden death	7.8%	13.1%	18.2%	24.6%	26.7%
ICD shock vs. non-sudden death					
	Low MAGGIC risk, *n* = 345				
Patients at risk	315	285	196	120	10
ICD shock	1.8%	2.1%	5.3%	7.7%	8.6%
Non-sudden death	2.7%	4.6%	6.4%	8.7%	9.5%
	Mid MAGGIC risk, *n* = 398				
Patients at risk	352	310	207	101	13
ICD shock	1.8%	2.9%	5.8%	8.8%	10.3%
Non-sudden death	5.2%	9.0%	10.9%	13.7%	14.6%
	High MAGGIC risk, *n* = 346				
Patients at risk	279	235	145	75	5
ICD shock	3.0%	4.9%	7.7%	9.1%	9.2%
Non-sudden death	10.8%	17.9%	24.0%	31.2%	33.5%

## Data Availability

Data can be made available through a reasonable request to the senior author.
